# Comparison of PKRP and TUVP in the treatment of high-risk BPH and analysis of postoperative influencing factors

**DOI:** 10.3389/fsurg.2022.947027

**Published:** 2022-08-03

**Authors:** Yao Song, Songqiang Pang, Gongtang Luo, Sen Li, Yaqiang He, Jinqiang Yang

**Affiliations:** Department of Urology Surgery, Shunyi Hospital, Beijing, China

**Keywords:** high-risk benign prostatic hyperplasia, transurethral plasmakinetic resection of the prostate, transurethral electrovaporization of the prostate, therapeutic effect, influencing factors

## Abstract

**Objective:**

This study aims to compare the efficacy of plasma kinetic loop resection of the prostate (PKRP) and transurethral vaporization of the prostate (TUVP) for the treatment of high-risk benign prostatic hyperplasia (BPH), and analyze the influence of the related factors on the operation of BPH.

**Methods:**

A total of 108 high-risk BPH patients diagnosed in our hospital from March 2018 to September 2021 were selected and randomly divided into an observation group and a control group, with 54 cases in each group. The control group was treated with TUVP, and the observation group was treated with PKRP. The international prostate symptom score (IPSS), quality of life (QOL) index, maximum urine flow rate (Qmax), and residual urine volume (RU) were observed before and after treatment. The general information such as age, educational level, residence, and residence status of the patient, as well as clinical information such as surgical method, nocturia frequency, preoperative IPSS score, RU, medical history, and prostate texture, were also recorded. All patients were followed up for 1 month, and complications were recorded.

**Results:**

The IPSS score, QOL score, and RU of patients in the two groups were lower after treatment than those before treatment, and the Qmax was higher than that before treatment (*P* < 0.05). The IPSS score, QOL score, and RU of the observation group were lower than those of the control group, and the Qmax was higher than that of the control group (*P* < 0.05). The incidence of postoperative complications in the observation group was lower than in the control group (*P* < 0.05). Univariate analysis showed that the patient's age, surgical method, nocturia frequency, preoperative IPSS score, RU, medical history, and prostatic texture all could affect the postoperative condition of patients with BPH (*P* < 0.05). Multivariate logistic analysis showed that the patient's age, surgical method, nocturia frequency, preoperative IPSS score, RU, and medical history were the independent influencing factors of the postoperative condition of patients with BPH (*P* < 0.05).

**Conclusion:**

PKRP in the treatment of high-risk BPH patients can effectively reduce the IPSS score, QOL score, and RU and significantly increase Qmax, with fewer complications and a good prognosis. Patients’ postoperative recovery was related to their age, surgical method, nocturia frequency, preoperative IPSS score, RU, and medical history. Therefore, choosing PKRP to treat high-risk BPH patients can effectively improve the postoperative urethral functional recovery of patients and reduce the occurrence of complications.

## Introduction

Benign prostatic hyperplasia (BPH) is a urological disease commonly occurring in middle-aged and elderly men, which manifests as lower urinary tract symptoms caused by enlarged prostate glands and outlet obstruction of the bladder neck, and seriously affects the quality of life of patients (1, 2). High-risk patients with BPH are often accompanied by basic diseases such as hypertension and diabetes. In addition, they are elderly and have poor body resistance, which greatly increases the difficulty of treatment (3, 4). At present, surgical treatment is commonly used in clinical practice. Transurethral resection of the prostate (TURP) is one of the main methods for the clinical treatment of BPH, but due to the inability to completely remove the gland tissue, the gland can still continue to proliferate and lower urinary tract symptoms appear again, which greatly impacts patients (5, 6). Transurethral vaporization of the prostate (TUVP) is an improved resection method based on TURP, which can effectively shorten the operation time and improve the resection quality. However, its effect on the improvement of postoperative urethral symptoms is limited (7, 8). Plasma kinetic loop resection of the prostate (PKRP) is a new type of prostatectomy, which is different from TURP in the working principle. A current does not need to pass through the body, but it can form a local control loop through normal saline to break the molecular bonds in the prostate tissue and thus destroy the tissue to relieve the symptoms of obstruction (9, 10). The purpose of this study was to compare the efficacy of PKRP and TUVP in the treatment of BPH and analyze the effects of relevant factors on the operation of BPH.

## Materials and methods

### Patients

A total of 108 patients with high-risk BPH diagnosed in our hospital from March 2018 to September 2021 were selected. Inclusion criteria are as follows: all of them met the guidelines for the diagnosis and treatment of benign prostatic hyperplasia (11); there were no contraindications of operation and use of anesthetic drugs; and patients with renal insufficiency did not improve significantly. Exclusion criteria are as follows: acute infection of the urinary system; patients with prostate cancer; patients with bladder stones and other diseases; patients with other additional serious organ diseases; patients with drug allergy; and patients who dropped out during follow-up. A total of 108 high-risk BPH patients were randomly divided into an observation group and a control group, with 54 cases in each group. There was no significant difference in general data between the two groups, as shown in Table 1.

**Table 1:** Comparison of general data between the two groups.

Group	Age (years)	Course of disease (years)	Preoperative IPSS score (points)
Control group (*n* = 54)	81.05 ± 6.21	4.94 ± 0.75	21.15 ± 2.56
Observation group (*n* = 54)	80.21 ± 6.52	5.08 ± 0.81	22.04 ± 2.75
*t*	0.679	0.932	1.741
*P*	0.498	0.354	0.085

### Surgical methods

Perioperative risk assessment was conducted before surgery, and surgery was arranged in the absence of absolute contraindication. If a urinary tract infection exists before surgery, empiric antibiotic treatment can be given, and a drug sensitivity test can be performed at the same time. Antibiotic medication can be adjusted according to the drug sensitivity test, urinary tract infection symptoms can be significantly improved, and surgical treatment can be performed later. If the patient has a history of urethral stricture, urethral dilation is feasible.

In both groups, the surgery was performed according to the standard procedures after lumbar anesthesia or continuous epidural anesthesia. The bladder lithotomy position was adopted, and the conventional bladder puncture fistulization was performed under television monitoring. The control group was treated with TUVP: a F26STORZ electrocision mirror (STORZ, Germany) was used, the power of vaporization electrocision was 200–230 W, the power of electrocoagulation was 80 W, and the content of lavage fluid was 5% mannitol. First, the hyperplastic glands were excised at 5–7 o’clock in advance and gradually cut in different regions. Finally, the periphery of the verruca was cut. With the bladder neck and the verruca as the marker points, the cutting depth is as deep as the surgical capsule as far as possible. The prostate fragment tissue was punched out by an Ellik evacuator, and the presence of the fragment residue was carefully examined again. F22 catheter was indwelled and bladder irrigation continued.

The observation group was treated with PKRP (GYRUS, UK). After successful anesthesia (continuous epidural block anesthesia was adopted for all patients), the lithotomy position was taken for the patient. The skin in the operation area was routinely sterilized with high-efficiency iodophor and then covered with a sterile towel. During the operation, the bladder was continuously rinsed, and amedical paraffin cotton ball was used to lubricate the electrocision lens sheath. After the lens sheath with the lens core was inserted, the lens core was extracted (if the external urethral orifice was relatively narrow, the urethral probe was used to expand and then the lens sheath was placed). An F26 resectoscope was placed along the sheath to observe the bladder and identify the location of the trigone of the bladder and bilateral ureterostoma, and then, the resectoscope was retreated to the posterior urethra to identify the prostatic hyperplasia and determine the location of the caruncle. The resection point was selected according to the location and degree of BPH. The bleeding was stopped by electric coagulation while the resection was performed. For obvious bilateral lobe hyperplasia, a landmark groove was cut at 6 o'clock to reach the level of the upper margin of Giemu, and the bilateral lobes were cut in sequence. If the hyperplasia of the middle lobe is more obvious, the 5:00 and 7: 00 positions should be marked first, and then the hyperplastic tissues of both lateral lobes and the middle lobe should be successively excised to the level of the upper margin of the Giemu to the depth of the prostatic capsule. Finally, the bladder neck and the prostatic apex were trimmed to ensure that the prostatic part of the urethra was a smooth tunnel. After careful electrocoagulation, hemostasis was performed on the whole wound surface and no active bleeding was detected. The endoscope was retracted. An Eric flusher was used to suck out the resected BPH tissue. An F20 or F22 three-cavity urinary catheter was retained, and the airbag was filled with water. The sterile oil yarn was used to tie a knot at the external orifice of the urethra. After a little traction and pressurization hemostasis were performed, the bladder was continuously rinsed with 0.9% sodium chloride isotonic rinse. And the operation was completed. The prostate tissue resected during the operation was sent for pathological examination for a definite diagnosis. After surgery, patients' consciousness and consciousness were closely monitored, ECG and pulse oxygen were monitored, and vital signs were closely monitored.

### Observation indicators

Intraoperative blood loss and hospital stay in the two groups were recorded. The international prostate symptom score (IPSS) (12), quality of life index (QOL) (13), maximum urine flow rate (Qmax), and residual urine volume (RU) were observed before and after treatment. The general information such as age, educational level, residence, and residence status of the patient, as well as clinical information such as surgical method, nocturia frequency, preoperative IPSS score, RU, medical history, and prostate texture, was also recorded. All patients were followed up for 1 month, and complications were recorded.

### Statistical methods

SPSS22.0 software was used for processing. The measurement data were expressed by mean ± standard deviation, and *t*-test analysis was used for pairwise comparisons. Count data were expressed by rate, and the chi-square test was used for the comparison between groups. A multivariate logistic regression model was used for multivariate analysis. *P* < 0.05 indicated that the difference was statistically significant.

## Results

### Comparison of intraoperative blood loss and hospital stay between two groups

The intraoperative blood loss and hospital stay in the observation group were lower than those in the control group (*P* < 0.05), as shown in Table 2.

**Table 2:** Comparison of intraoperative blood loss and hospital stay between two groups.

Group	Intraoperative blood loss (mL)	Hospital stay (d)
Control group	348.52 ± 70.48	7.75 ± 2.58
Observation group	210.54 ± 60.46	6.42 ± 1.98
*t*	10.919	3.005
*P*	<0.001	0.003

### Comparison of various efficacy indicators between the two groups

**Figure 1:**
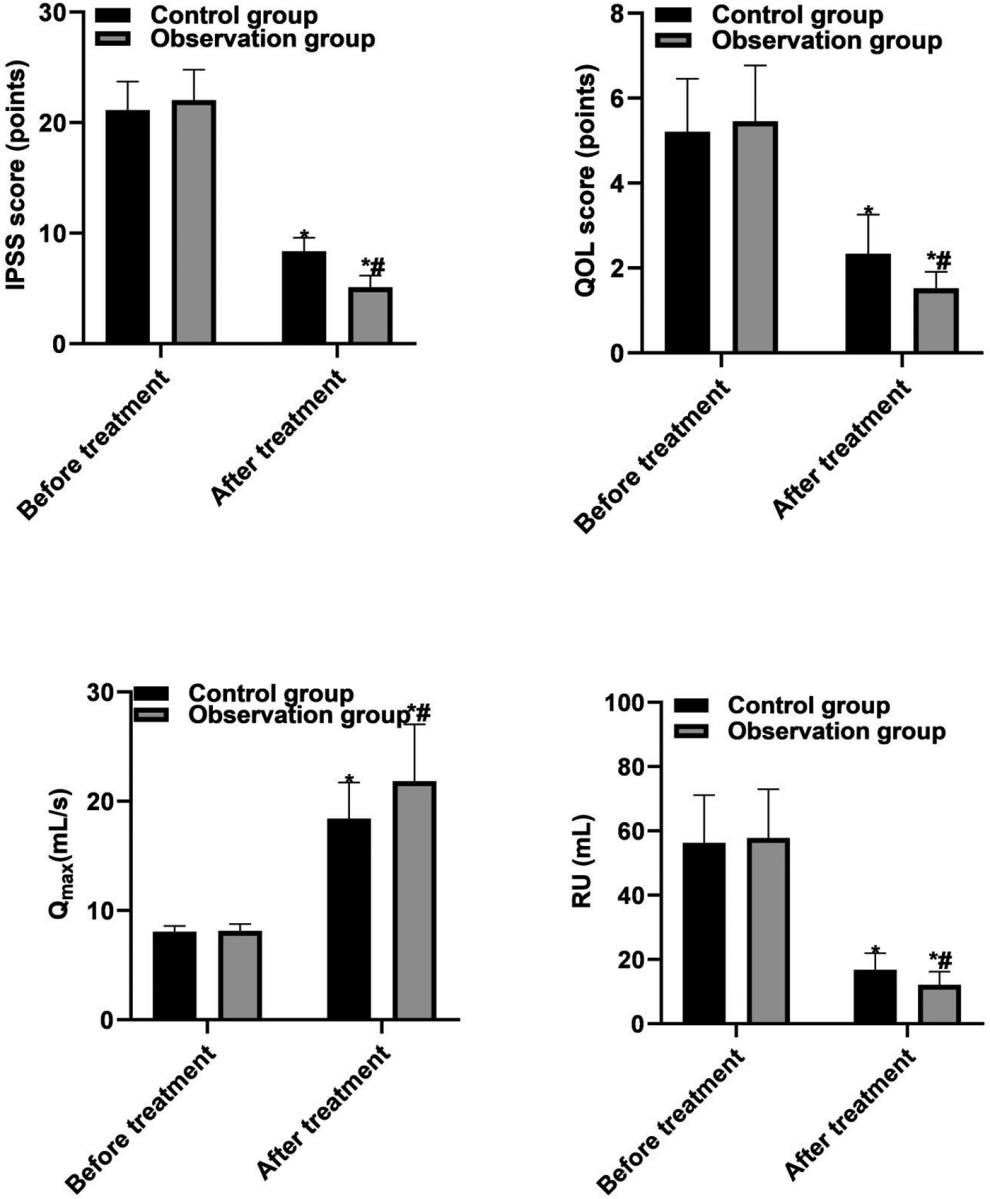
Comparison of various efficacy indicators between the two groups. Note: Compared with before treatment, *P < 0.05; compared with the control group, ^#^*P* < 0.05.

The IPSS score, QOL score, and RU of the two groups of patients after treatment were lower than those before treatment, and the Qmax was higher than that before treatment (*P* < 0.05). After the treatment, the IPSS score, QOL score, and RU of the observation group were lower than those of the control group, and the Qmax was higher than that of the control group (*P* < 0.05), as shown in Figure 1.

### Postoperative complications in two groups

**Figure 2:**
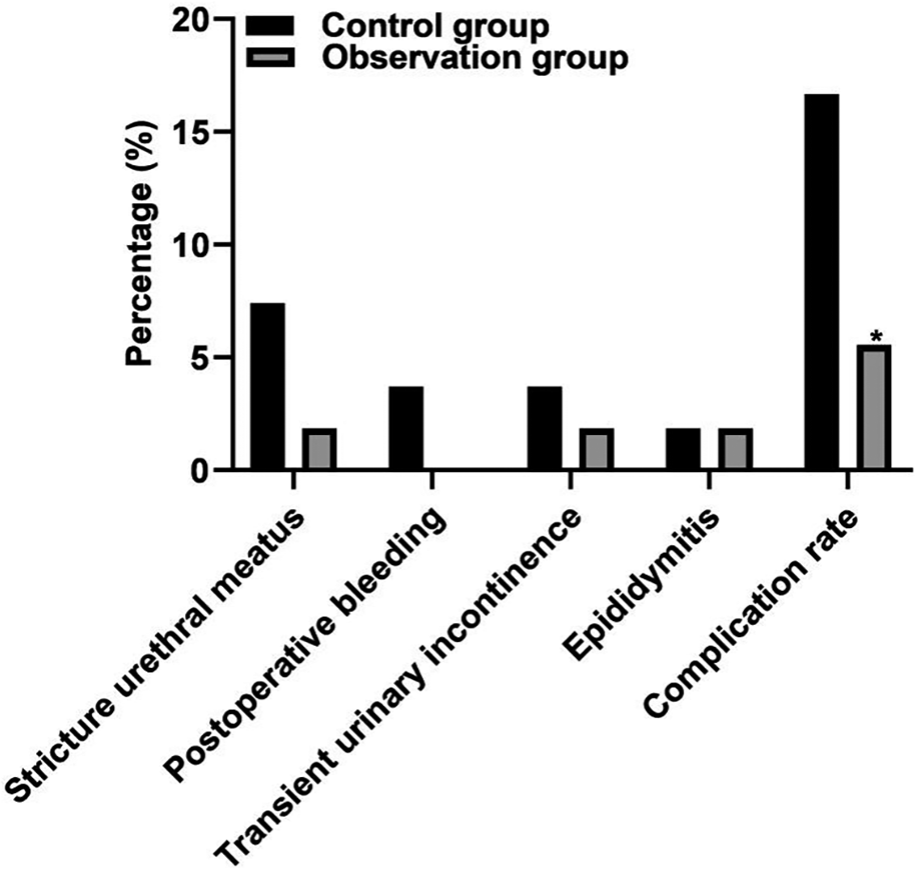
Postoperative complications in two groups. Note: Compared with the control group, *P < 0.05.

In the control group, urethral stricture was found in four cases, postoperative hemorrhage in two cases, transient urinary incontinence in two cases, and epididymitis in one case. The complication rate was 16.67% (9/54). In the observation group, there was one case of urethral stricture, one case of transient urinary incontinence, and one case of epididymitis, and the incidence of complications was 5.56% (3/54). There was a significant difference in the incidence of complications between the two groups (*P* < 0.05), as shown in Figure 2.

### Single-factor analysis of the postoperative condition (IPSS score) of patients with BPH

Univariate analysis showed that the patient's age, surgical method, nocturia frequency, preoperative IPSS score, RU, medical history, and prostatic texture all could affect the postoperative condition of patients with BPH (*P* < 0.05), as shown in Table 3.

**Table 3:** Univariate analysis of the postoperative condition (IPSS score) of BPH patients.

Influencing factor		Cases	Postoperative IPSS score (score)	*t*	*P*
Age (years)	70–79	50	5.03 ± 1.86	4.896	0.041
	80–89	58	7.89 ± 2.28		
Degree of education	Junior secondary and below	33	6.55 ± 2.55	1.208	0.462
	High school and above	75	6.75 ± 2.31		
Place of residence	village	58	6.62 ± 2.06	1.606	0.368
	cities and towns	50	6.77 ± 2.44		
Living conditions	live in solitude	32	6.59 ± 2.33	1.385	0.405
	Not living alone	76	6.95 ± 1.59		
Surgical methods	TUVP	54	8.36 ± 1.22	5.472	0.033
	PKRP	54	5.12 ± 1.03		
Number of nocturia (times)	<3	25	5.89 ± 1.92	4.208	0.046
	≥3	83	7.28 ± 2.65		
Preoperative IPSS score (points)	0–10	12	4.08 ± 0.83	8.056	0.001
	11–20	40	7.05 ± 1.22		
	21–30	56	12.52 ± 2.84		
RU (mL)	≥60	70	8.26 ± 2.58	4.586	0.042
	<60	38	5.22 ± 1.77		
Medical history (years)	0–3	23	4.25 ± 0.93	7.589	0.006
	3–5	33	8.18 ± 1.35		
	>5	52	11.98 ± 2.06		
Prostate texture	soft	14	5.06 ± 1.18	6.872	0.012
	middle	67	7.68 ± 2.15		
	hard	27	11.24 ± 2.91		

### Multifactor analysis of the postoperative condition (IPSS score) of patients with BPH

Multivariate logistic analysis showed that the patient's age, surgical method, nocturia frequency, preoperative IPSS score, RU, and medical history were the independent influencing factors of the postoperative condition of patients with BPH (*P* < 0.05), as shown in Tables 4, 5.

**Table 4:** Multifactor analysis assignment table.

Factors	Variable	Assignment
Age	X1	70–79 = 0, 80–89 = 1
Surgical methods	X2	TUVP = 0, PKRP = 1
Number of nocturia	X3	<3 = 0, ≥3 = 1
Preoperative IPSS score	X4	0–10 = 0, 11–20 = 1, 21–30 = 2
RU	X5	≥60 = 0, <60 = 1
Medical history	X6	0–3 = 0, 3–5 = 1, >5 = 2
Prostate texture	X7	soft = 0, middle = 1, hard = 2

**Table 5:** Multifactor analysis of the postoperative condition (IPSS score) of patients with BPH.

Influencing factor	B	SE	Walds	*P*	OR	95% CI
Age	0.851	0.533	5.102	0.038	1.533	1.049–1.996
Surgical methods	0.921	0.594	7.025	0.009	1.991	1.405–2.554
Number of nocturia	1.106	0.756	4.693	0.042	1.728	1.372–2.164
Preoperative IPSS score	0.786	0.642	5.955	0.029	2.159	1.298–3.052
RU	1.322	0.495	4.524	0.044	2.385	1.637–2.983
Medical history	0.754	0.518	6.875	0.019	1.958	1.097–3.104
Prostate texture	0.163	0.215	1.833	0.084	1.215	0.896–1.632

## Discussion

Patients with BPH are mainly men over 40 years old. The main symptoms of BPH are enlarged prostate glands and a blocked bladder outlet, causing lower urinary tract symptoms and even possibly leading to renal dysfunction. Especially for high-risk BPH patients, their older age, poorer body function, and more basic diseases increase the difficulty of treatment (14, 15). The treatment of BPH currently mainly includes surgical treatment and nonsurgical treatment. Nonoperative treatment is mainly performed through drugs. Drug treatment is mainly applied to mild lower urinary tract (LUT) symptoms caused by BPH. Most of the LUT symptoms are in the early stage of disease development. LUT symptoms exist but have not yet seriously affected daily life. Such patients can relieve LUT symptoms, delay disease development, inhibit bladder overactivity, promote urination, protect kidney function, prevent and avoid hematuria through drug treatment, and can also be given antibiotics to control urinary system infection and reduce the occurrence of acute urinary retention (16, 17). When the symptoms of LUT are serious, they can seriously affect and interfere with the daily life of patients; Or only have mild LUTs symptoms and have received drug treatment, but the symptoms are not significantly relieved or even worsened, the patient's subjective tolerance is poor, and the patient is seriously troubled; Or repeated hematuria, multiple urinary system infections, urinary retention cannot be alleviated, bladder stones appear, and secondary upper urinary tract hydronephrosis appear (18, 19). TURP is still the gold standard for the treatment of BPH, but it still has certain limitations in practice. High-risk prostatic hyperplasia is considered the relative contraindication for TURP surgery. Most patients can only receive palliative indwelling urinary catheter, cystostomy, and medication, resulting in poor quality of life (20, 21). Therefore, it is extremely important to select effective treatment methods for high-risk BPH patients.

The results of this study showed that intraoperative blood loss and hospital stay in the observation group were lower than those in the control group, indicating that PKRP could effectively reduce the intraoperative blood loss and hospital stay. The results of this study showed that the IPSS score, QOL score, and RU of patients in the two groups after treatment were lower than those before treatment, and Qmax was higher than that before treatment. The IPSS score, QOL score, and RU of patients in the observation group were lower than those of patients in the control group, and Qmax was higher than that of patients in the control group. One of the reasons was that the working principles of PKRP were different from those of TUVP. The working current of PKRP did not need to pass through the body but could directly pass through normal saline to form a local control loop, breaking the molecular bonds in the prostate tissue to destroy the tissue and relieve the symptoms of obstruction, which effectively reduced the tissue damage and improved the symptoms of postoperative urethral stimulation (22, 23). In addition, the research results show that the incidence of postoperative complications in the observation group is significantly lower than that in the control group. Urethral stricture may be caused by a urinary tract infection. The preoperative urinary tract infection is not completely controlled, the preoperative examination and surgical instruments are not disinfected thoroughly, the urethral mucosa is damaged due to lithotripsy and stone removal during the operation for the patients who are accompanied by bladder stones, the postoperative anti-infection treatment and perioperative nursing care were insufficient, and so on. All these aggravate the edema of local tissues, prolong the wound healing time, and finally lead to wound fibrosis and hyperplasia, and then scar healing, the formation of urethral stenosis (24, 25). Urethral stricture may also be caused by preoperative or intraoperative urethral dilatation. When the lens sheath is not sufficiently lubricated, it can also cause damage to the urethral mucosa during the insertion of the lens sheath, and in addition, the long-time compression of the electrotomy lens sheath can lead to ischemia, necrosis, fibrotic hyperplasia, and cicatrix healing of local tissue mucosa, finally forming urethral stricture. Stenosis caused by iatrogenic injury is usually caused by ischemia following uroendoscopic surgery or long-term indwelling catheter. During catheterization, the operator did not act gently enough and the catheter model was thick, hard, and not lubricated enough, thus damaging the urethral wall. Indwelling the postoperative catheter for a long time and long-term compression of the urethral wall lead to local tissue mucosal edema, ischemic necrosis scar healing, and then formation of urethral meatus stenosis (26, 27).

The results of this study show that the patient's age, surgical method, nocturia frequency, preoperative IPSS score, RU, medical history, and prostate texture can all affect the postoperative condition of patients with BPH. Multivariate logistic analysis showed that the patient's age, surgical method, nocturia frequency, preoperative IPSS score, RU, and medical history were the independent influencing factors of the postoperative condition in patients with BPH. The reasons were analyzed as follows: As patients get older, their body immunity weakens and their tolerance to large-scale surgery weakens. Moreover, elderly patients often suffer from chronic diseases such as hypertension, which greatly affects their recovery after surgery. PKRP and TUVP work on different principles. Current does not need to pass through the body, causing little damage to the prostate tissue. Moreover, due to the clear surgical field, the prostate tissue can be excised more accurately, which is conducive to the patient's postoperative urethral recovery. For patients with severe disease and a long history of prostate hyperplasia, it is difficult to resect the prostate tissue during the operation, so it is very easy to affect the curative effect of the operation.

## Conclusion

PKRP in the treatment of high-risk BPH patients can effectively reduce the IPSS score, QOL score, and RU and significantly increase Qmax, with fewer complications and a good prognosis. Postoperative recovery was related to the patients’ age, nocturia frequency, preoperative IPSS score, RU, and medical history, and the surgical method used. Therefore, selecting PKRP for the treatment of high-risk BPH patients can effectively improve the postoperative urethral functional recovery of patients and reduce the occurrence of complications.

## Data Availability

The original contributions presented in the study are included in the article/Suplementary Material, further inquiries can be directed to the corresponding author/s.
